# Supplementary material for: Role of mutational reversions and fitness restoration in Zika virus spread to the Americas
Source: Nat Commun. 2021 Jan 26;12:595. doi: 10.1038/s41467-020-20747-3 (PMC7838395; doi:10.1038/s41467-020-20747-3)
Supplement: Supplementary file 1 — Supplementary Information [file 41467_2020_20747_MOESM1_ESM.pdf]

Role of mutational reversions and fitness restoration in Zika virus spread to the Americas

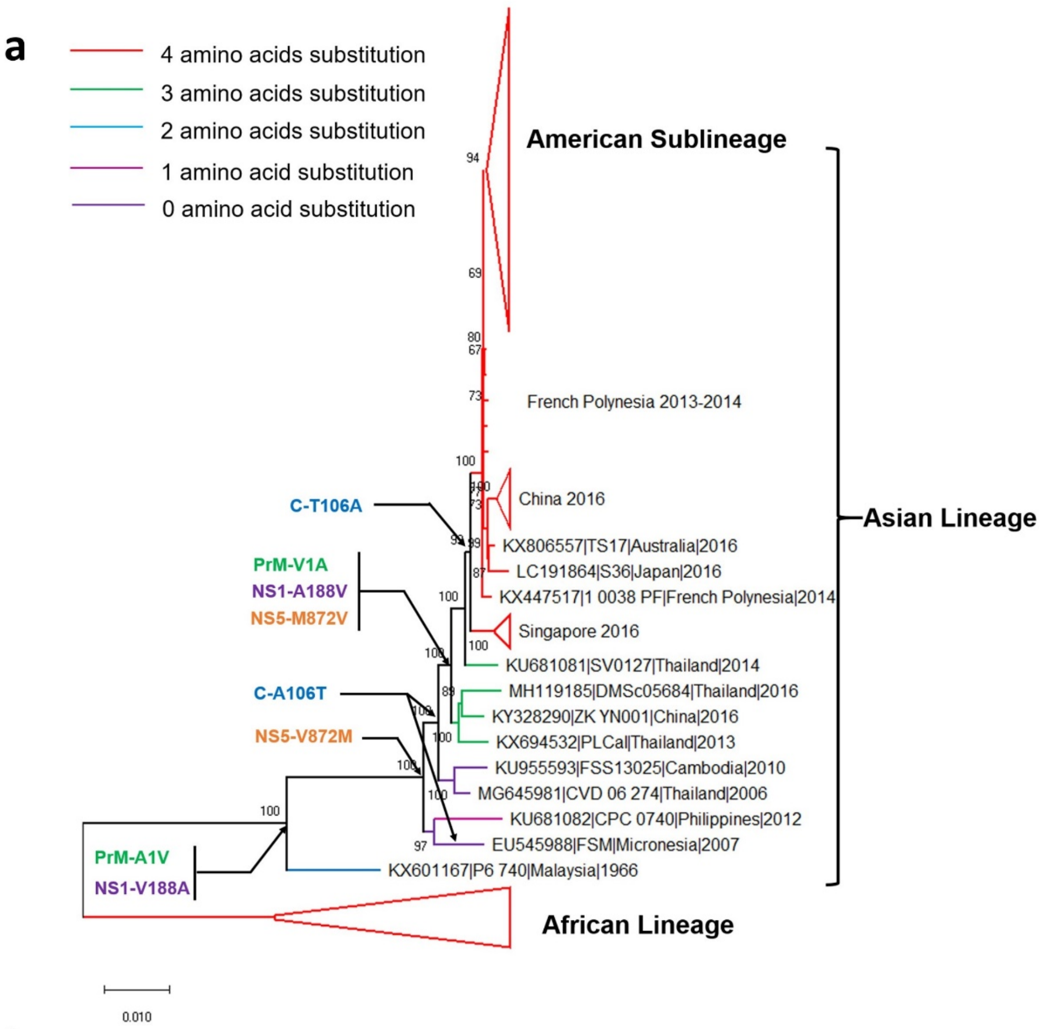

b

Position		African Lineage		Asian Lineage (Pre-epidemic)		Asian Lineage (American)	
Gene	Amino acid	Codon	Amino acid	Codon	Amino acid	Codon	Amino acid
**Capsid**	106	GCT	A	ACA	T	GCA	A
**PrM**	1	GCC	A	GTG	V	GCG	A
**NS1**	188	GTC	V	GCT	A	GTT	V
**NS5**	872	GTG	V	ATG	M	GTG	V

Supplementary Fig. 1. Phylogenetic analysis of representative ZIKV strains.

a, Phylogenetic tree of representative Zika virus (ZIKV) strains based on 73 complete open reading frame sequences. The evolutionary distances were computed using the Maximum Composite Likelihood method. The percentage of replicate trees in which the associated taxa clustered together in the bootstrap tests (1000 replicates) were shown next to the branches, except for those with values of 100 or <50. Colors of branches show strains and hypothetical ancestors with 0-4 of the reversions shown in the branch labels (C-T106A, prM-V1A, NS1-A188V, NS5-M872V). Evolutionary analyses were performed in MEGA X, by using the Maximum Likelihood method. **b**, Four reversion directly reverting mutations compared among representative African, Asian and American lineage strains.

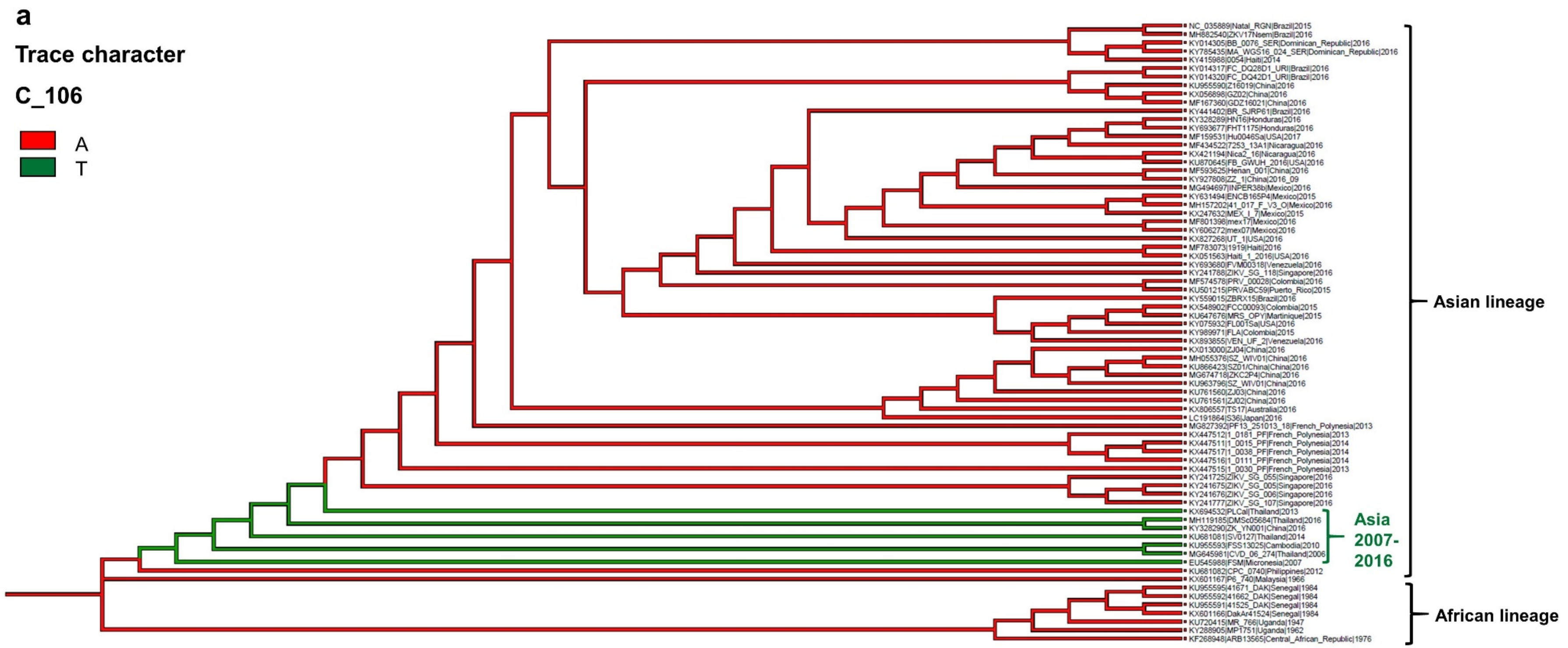
a
Trace character
C_106
A
T
Asian lineage
Asia 2007-2016
African lineage

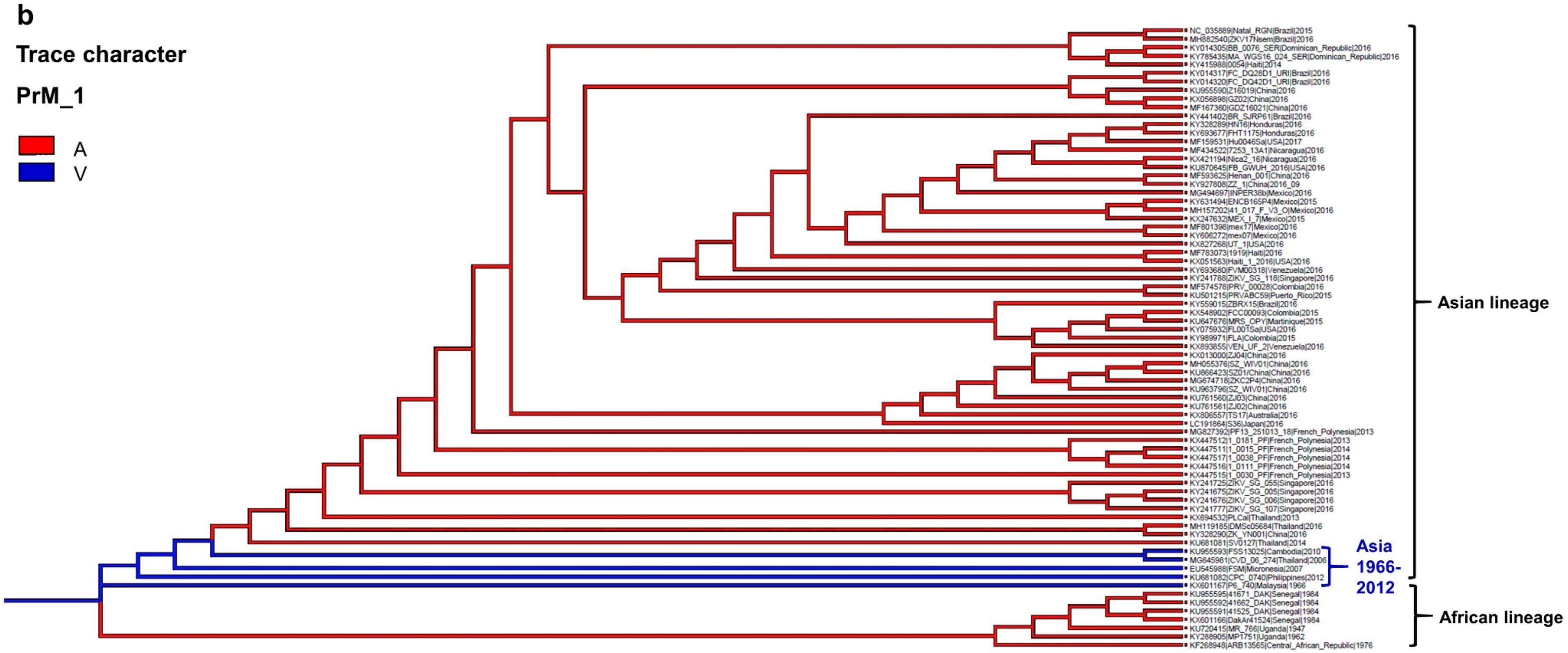
b
Trace character
PrM_1
A
V
NC_035889|Natal_RGN|Brazil|2015
MH882540|ZKV17Nsem|Brazil|2016
KY014305|BB_0076_SER|Dominican_Republic|2016
KY785435|MA_WGS16_024_SER|Dominican_Republic|2016
KY415988|0054|Haiti|2014
KY014317|FC_DQ28D1_URI|Brazil|2016
KY014320|FC_DQ42D1_URI|Brazil|2016
KU955590|Z16019|China|2016
KX056898|GZ02|China|2016
MF167360|GDZ16021|China|2016
KY441402|BR_SJRP61|Brazil|2016
KY328289|HN16|Honduras|2016
KY693677|FHT1175|Honduras|2016
MF159531|Hu0046Sa|USA|2017
MF434522|7253_13A1|Nicaragua|2016
KX421194|Nica2_16|Nicaragua|2016
KU870645|FB_GWUH_2016|USA|2016
MF593625|Henan_001|China|2016
KY927808|ZZ_1|China|2016_09
MG494697|INPER38b|Mexico|2016
KY631494|ENCB165P4|Mexico|2015
MH157202|41_017_F_V3_O|Mexico|2016
KX247632|MEX_I_7|Mexico|2015
MF801398|mex17|Mexico|2016
KY606272|mex07|Mexico|2016
KX827268|UT_1|USA|2016
MF783073|1919|Haiti|2016
KX051563|Haiti_1_2016|USA|2016
KY693680|FVM00318|Venezuela|2016
KY241788|ZIKV_SG_118|Singapore|2016
MF574578|PRV_00028|Colombia|2016
KU501215|PRVABC59|Puerto_Rico|2015
KY559015|ZBRX15|Brazil|2016
KX548902|FCC00093|Colombia|2015
KU647676|MRS_OPY|Martinique|2015
KY075932|FL001Sa|USA|2016
KY989971|FLA|Colombia|2015
KX893855|VEN_UF_2|Venezuela|2016
KX013000|ZJ04|China|2016
MH055376|SZ_WIV01|China|2016
KU866423|SZ01/China|China|2016
MG674718|ZKC2P4|China|2016
KU963796|SZ_WIV01|China|2016
KU761560|ZJ03|China|2016
KU761561|ZJ02|China|2016
KX806557|TS17|Australia|2016
LC191864|S36|Japan|2016
MG827392|PF13_251013_18|French_Polynesia|2013
KX447512|1_0181_PF|French_Polynesia|2013
KX447511|1_0015_PF|French_Polynesia|2014
KX447517|1_0038_PF|French_Polynesia|2014
KX447516|1_0111_PF|French_Polynesia|2014
KX447515|1_0030_PF|French_Polynesia|2013
KY241725|ZIKV_SG_055|Singapore|2016
KY241675|ZIKV_SG_005|Singapore|2016
KY241676|ZIKV_SG_006|Singapore|2016
KY241777|ZIKV_SG_107|Singapore|2016
KX694532|PLCal|Thailand|2013
MH119185|DMSc05684|Thailand|2016
KY328290|ZK_YN001|China|2016
KU681081|SV0127|Thailand|2014
KU955593|FSS13025|Cambodia|2010
MG645981|CVD_06_274|Thailand|2006
EU545988|FSM|Micronesia|2007
KU681082|CPC_0740|Philippines|2012
KX601167|P6_740|Malaysia|1966
KU955595|41671_DAK|Senegal|1984
KU955592|41662_DAK|Senegal|1984
KU955591|41525_DAK|Senegal|1984
KX601166|DakAr41524|Senegal|1984
KU720415|MR_766|Uganda|1947
KY288905|MP1751|Uganda|1962
KF268948|ARB13565|Central_African_Republic|1976
Asian lineage
Asia 1966-2012
African lineage

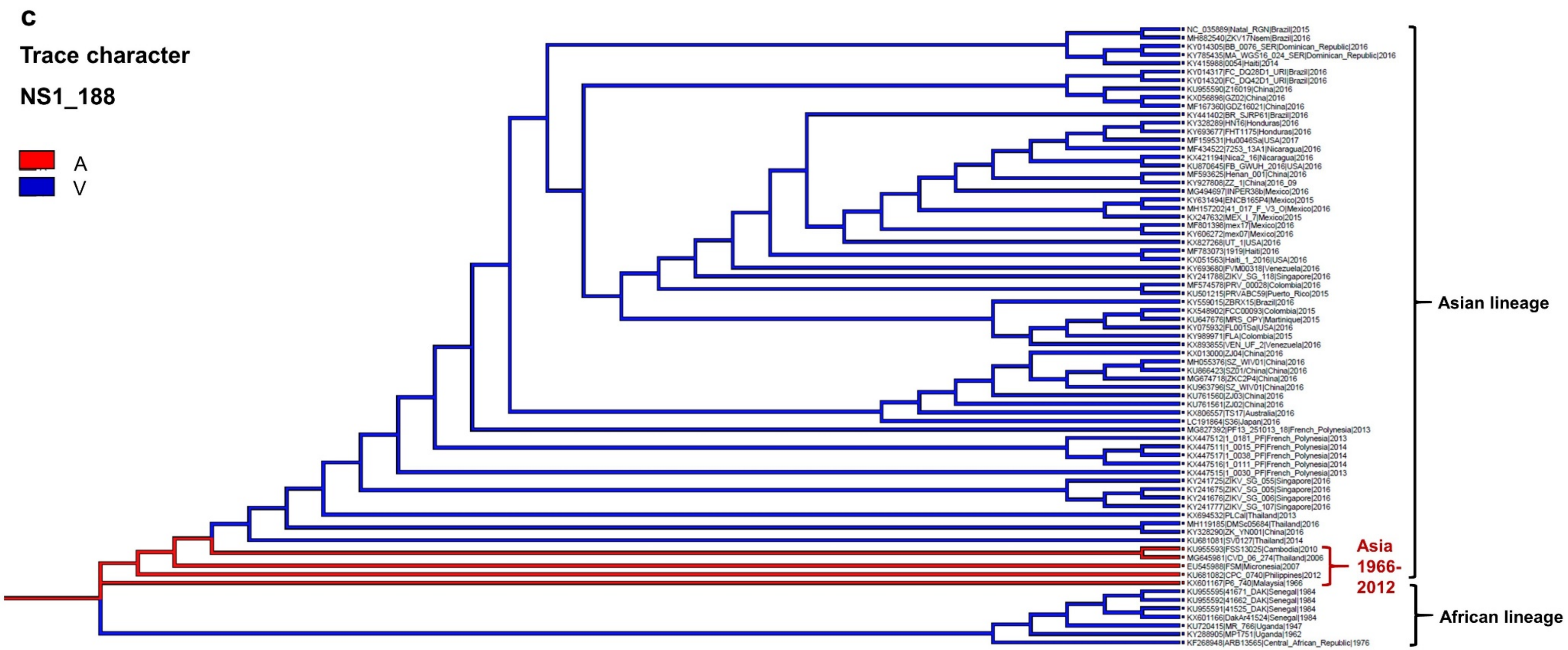
c
Trace character
NS1_188
A
V
NC_035889|Natal_RGN|Brazil|2015
MH882540|ZKV17Nsem|Brazil|2016
KY014305|BB_0076_SER|Dominican_Republic|2016
KY785435|MA_WGS16_024_SER|Dominican_Republic|2016
KY415988|0054|Haiti|2014
KY014317|FC_DQ28D1_URI|Brazil|2016
KY014320|FC_DQ42D1_URI|Brazil|2016
KU955590|Z16019|China|2016
KX056898|GZ02|China|2016
MF167360|GDZ16021|China|2016
KY441402|BR_SJRP61|Brazil|2016
KY328289|HN16|Honduras|2016
KY693677|FHT1175|Honduras|2016
MF159531|Hu0046Sa|USA|2017
MF434522|7253_13A1|Nicaragua|2016
KX421194|Nica2_16|Nicaragua|2016
KU870645|FB_GWUH_2016|USA|2016
MF593625|Henan_001|China|2016
KY927808|ZZ_1|China|2016_09
MG494697|INPER38b|Mexico|2016
KY631494|ENCB165P4|Mexico|2015
MH157202|41_017_F_V3_O|Mexico|2016
KX247632|MEX_I_7|Mexico|2015
MF801398|mex17|Mexico|2016
KY606272|mex07|Mexico|2016
KX827268|UT_1|USA|2016
MF783073|1919|Haiti|2016
KX051563|Haiti_1_2016|USA|2016
KY693680|FVM00318|Venezuela|2016
KY241788|ZIKV_SG_118|Singapore|2016
MF574578|PRV_00028|Colombia|2016
KU501215|PRVABC59|Puerto_Rico|2015
KY559015|ZBRX15|Brazil|2016
KX548902|FCC00093|Colombia|2015
KU647676|MRS_OPY|Martinique|2015
KY075932|FL001Sa|USA|2016
KY989971|FLA|Colombia|2015
KX893855|VEN_UF_2|Venezuela|2016
KX013000|ZJ04|China|2016
MH055376|SZ_WIV01|China|2016
KU866423|SZ01/China|China|2016
MG674718|ZKC2P4|China|2016
KU963796|SZ_WIV01|China|2016
KU761560|ZJ03|China|2016
KU761561|ZJ02|China|2016
KX806557|TS17|Australia|2016
LC191864|S36|Japan|2016
MG827392|PF13_251013_18|French_Polynesia|2013
KX447512|1_0181_PF|French_Polynesia|2013
KX447511|1_0015_PF|French_Polynesia|2014
KX447517|1_0038_PF|French_Polynesia|2014
KX447516|1_0111_PF|French_Polynesia|2014
KX447515|1_0030_PF|French_Polynesia|2013
KY241725|ZIKV_SG_055|Singapore|2016
KY241675|ZIKV_SG_005|Singapore|2016
KY241676|ZIKV_SG_006|Singapore|2016
KY241777|ZIKV_SG_107|Singapore|2016
KX694532|PLCal|Thailand|2013
MH119185|DMSc05684|Thailand|2016
KY328290|ZK_YN001|China|2016
KU681081|SV0127|Thailand|2014
KU955593|FSS13025|Cambodia|2010
MG645981|CVD_06_274|Thailand|2006
EU545988|FSM|Micronesia|2007
KU681082|CPC_0740|Philippines|2012
KX601167|P6_740|Malaysia|1966
KU955595|41671_DAK|Senegal|1984
KU955592|41662_DAK|Senegal|1984
KU955591|41525_DAK|Senegal|1984
KX601166|DakAr41524|Senegal|1984
KU720415|MR_766|Uganda|1947
KY288905|MP1751|Uganda|1962
KF268948|ARB13565|Central_African_Republic|1976
Asian lineage
Asia
1966-
2012
African lineage

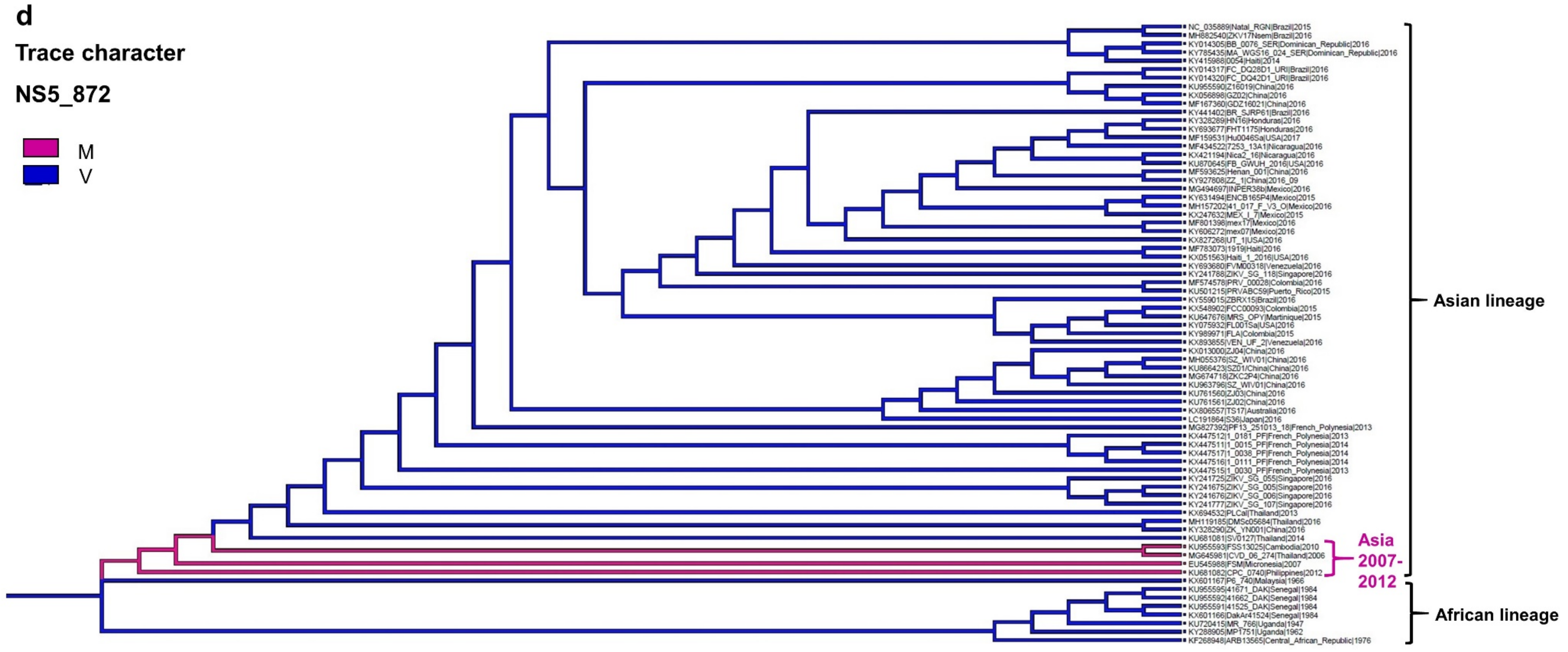

Supplementary Fig. 2. The trace history of four directly reverting amino acids in the evolutionary tree of Zika virus.

Trace history of four ZIKV amino acids performed in Mesquite (Version 3.61, http://www.mesquiteproject.org). These include **a**. Amino acid 106 substitution of the capsid protein; **b**. Amino acid 1 of the pre-membrane (prM) protein; **c**. amino acid 188 of the nonstructural protein 1 (NS1), and; **d**. amino acid 872 of the nonstructural protein 5 (NS5) based on 73 complete zika virus open reading frame sequences.

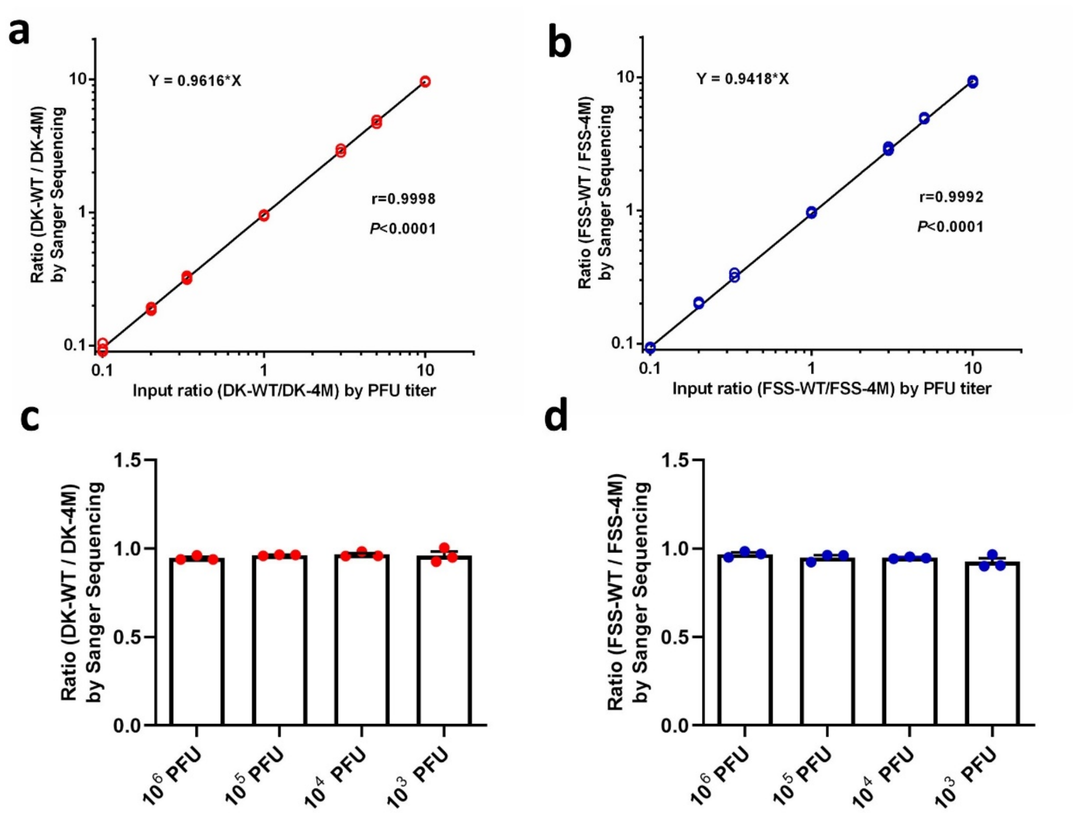

Supplementary Fig. 3. The consistency and accuracy validation of competition assay by Sanger sequencing.

a, **b**, The correlation between PFU input ratios and output ratios determined by Sanger sequencing of RT-PCR amplicons. The DK-WT/DK-4M (**a**) or FSS-WT/FSS-4M(**b**) ZIKVs were mixed at different ratios of 10:1, 5:1, 3:1, 1:1, 1:3, 1:5, 1:10 based on the PFU titer. The total RNA of these mixed virus was isolated and transcribed by RT-PCR. The ratios of DK-WT/DK-4M and FSS-WT/FSS-4M were calculated based on the peak heights by Sanger sequencing. Data were analyzed by linear regression with correlation coefficients (r) and significance (p). **c**, **d**, The ratio of wt/mutant virus mixture calculated by Sanger sequencing was consistent when using virus mixture that ranges from high to low titer. The DK-WT/DK-4M (**c**) or FSS-WT/FSS-4M(**d**) ZIKVs were mixed at a PFU ratio of 1:1. The total titers of the mixed viruses were 10^6, 10^5, 10^4 and 10^3 PFU. The total RNA of these mixed virus was isolated and transcribed by RT-PCR. The ratios of DK-WT/DK-4M and FSS-WT/FSS-4M were calculated based on the peak heights by Sanger sequencing. Symbols represent individual samples, bar heights represent means, and error bars represent stand error of mean (SEM). **a-d**, n=3 biologically independent samples from one single experiment. Source data are provided as a Source Data file.

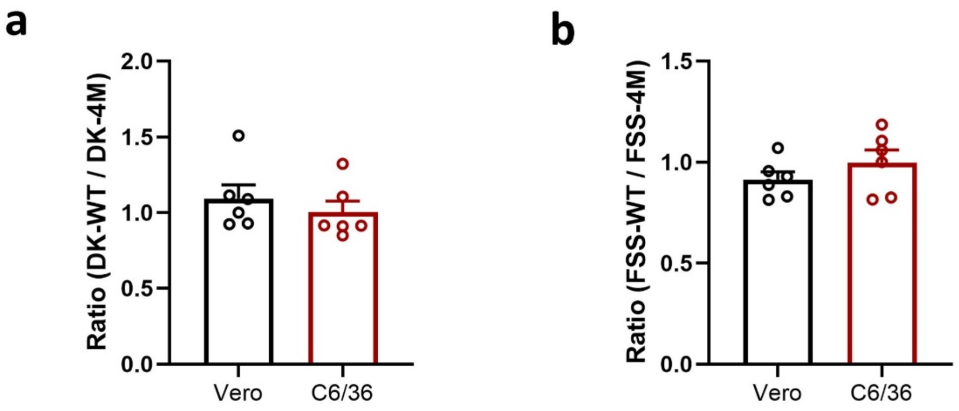

Supplementary Fig. 4. ZIKV WT and 4M mutant strain had a similar initial ratio when titrated on mammalian or insect cells.

a, **b**, The initial ratio of DK-WT/DK-4M (**a**) and FSS-WT/FSS-4M (**b**) were calculated by the virus titer determined on Vero and C6/C36 cells. The titers of the WT and mutant viruses were determined by FFA (Focus-forming assay) on the cells before they were mixed. Symbols represent individual samples, bar heights represent means, and error bars represent stand error of mean (SEM). n=6 biologically independent samples from 2 independent repeats. Source data are provided as a Source Data file.

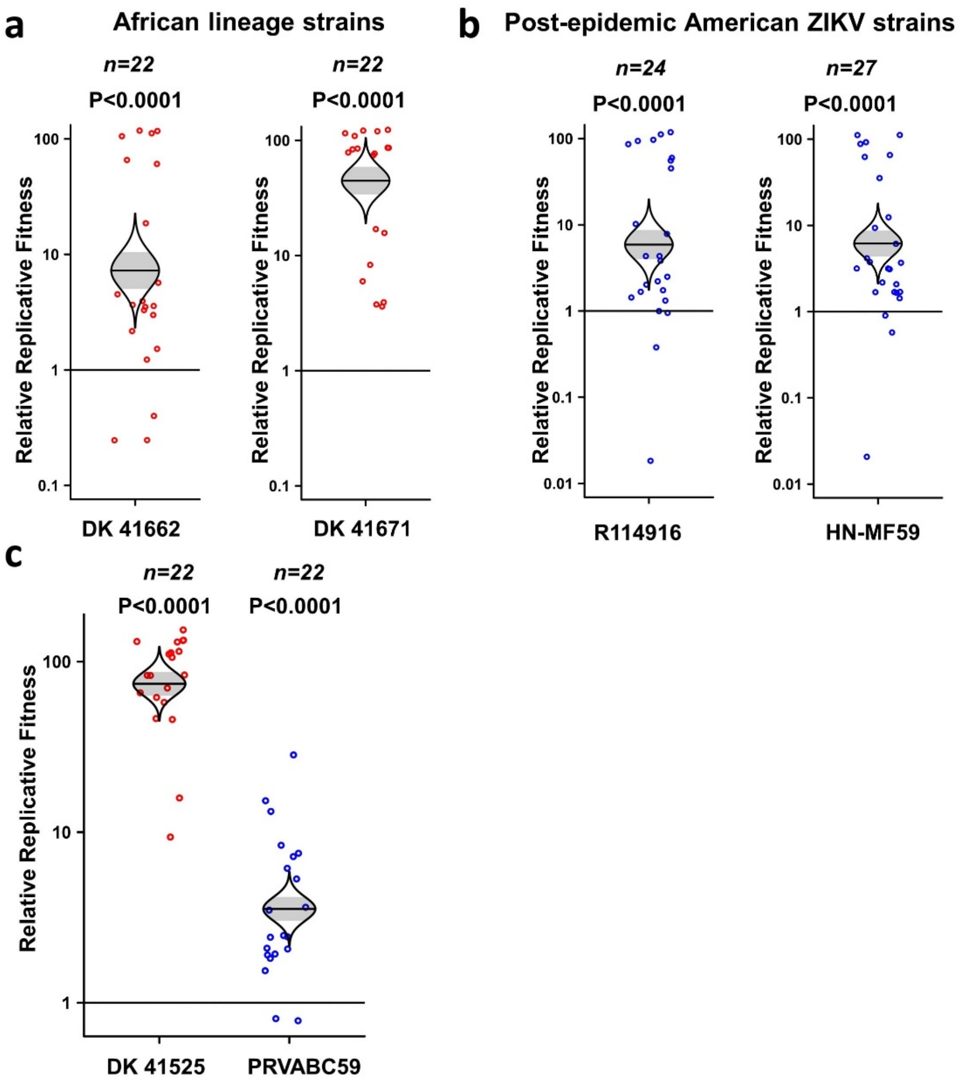

Supplementary Fig. 5. Fitness comparisons with additional Zika virus strains Dominican Republic *Aedes aegypti* mosquitoes.

a, The fitness of 2 African ZIKV strains (DK 41662) and (DK 41671) in competition with the Asian FSS13025 strain in mosquitoes. **b**, The fitness of 2 American ZIKV strains (R114916) and (HN-MF59) in competition with the FSS13025 Asian strain in mosquitoes. **c.** The fitness of ZIKV African strain Dakar 41525, Asian pre-epidemic strain FSS13025 strain and American strain PRVABC59 in a low generation (F6) colonized *A. aegypti* strain from the Dominican Republic. Each point represents a single mosquito or mouse sample **a-c**, The distribution of the model-adjusted means is illustrated by catseye plots with shaded +/- standard error overlaid by scatterplots of subject measures; scatterplots have been randomly jittered horizontally for clarity, and are shown on the log (base-10) scale such that comparisons are against a null value of 1. *P* values are calculated for the group (strain) coefficient for each linear regression model. *n* numbers represent the biologically independent samples and shown on the top of each figure. The results were pooled from 2 independent repeats. Source data are provided as a Source Data file.

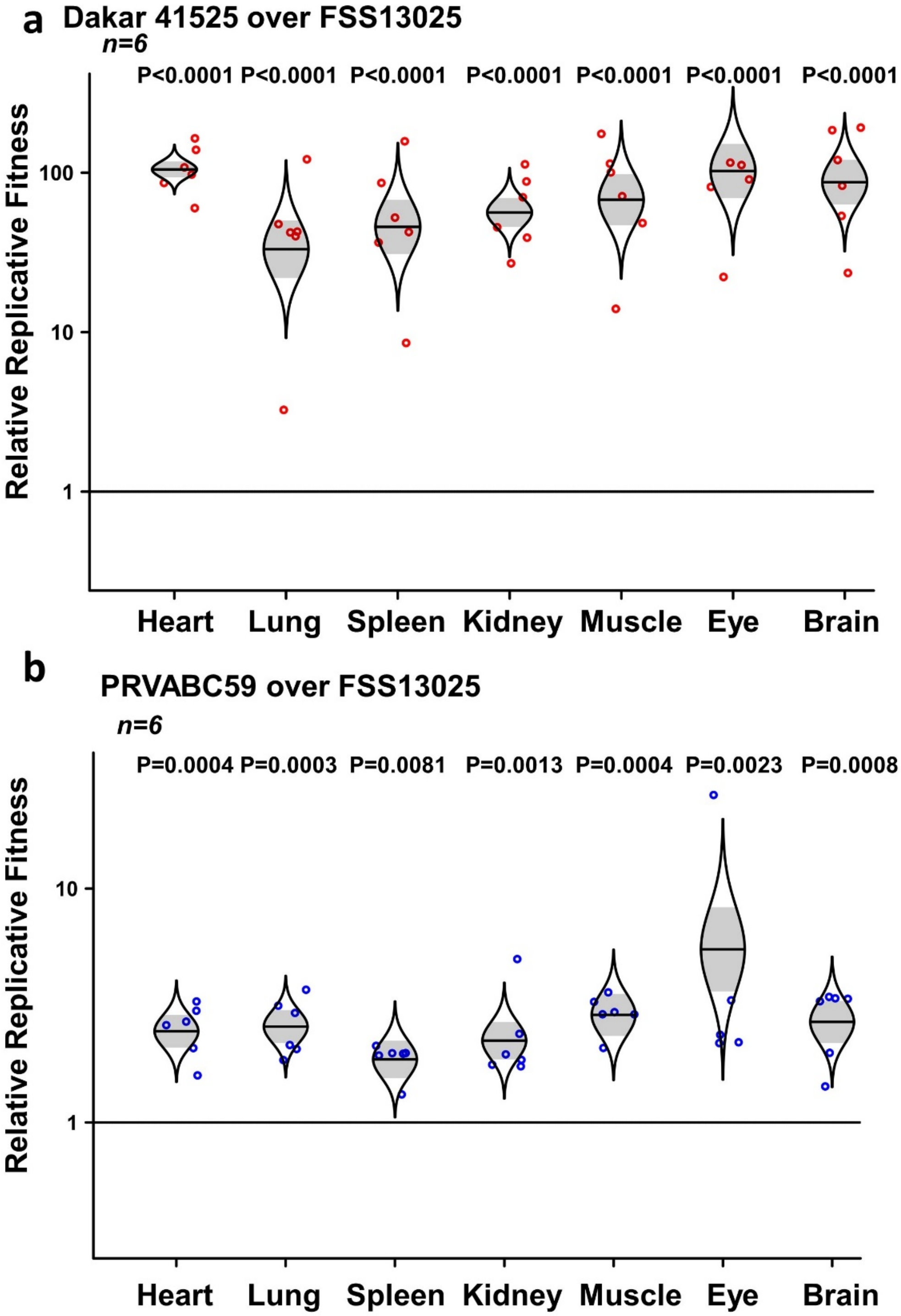

Supplementary Fig. 6. Fitness comparisons of African and post-epidemic against a pre-epidemic ZIKV strains in different organs of A129 mice.

a, Fitness comparison between African (Dakar 41525) and Asian pre-epidemic (FSS13025) ZIKV strains in different organs of A129 mice after 8 days of infection, when viremia had ended. **b**, The fitness comparison between Asian pre-epidemic (FSS13025) and American (PRVABC59) ZIKV strains in different organs of A129 mice

after 8 days of infection, when viremia had ended. Each point represents a single mouse sample **a**, **b**, The distribution of the model-adjusted means is illustrated by catseye plots with shaded +/- standard error overlaid by scatterplots of subject measures; scatterplots have been randomly jittered horizontally for clarity, and are shown on the log (base-10) scale such that comparisons are against a null value of 1. *P* values are calculated for the group (strain) coefficient for each linear regression model. *n*=6 biologically independent samples from a single experiment. Source data are provided as a Source Data file.

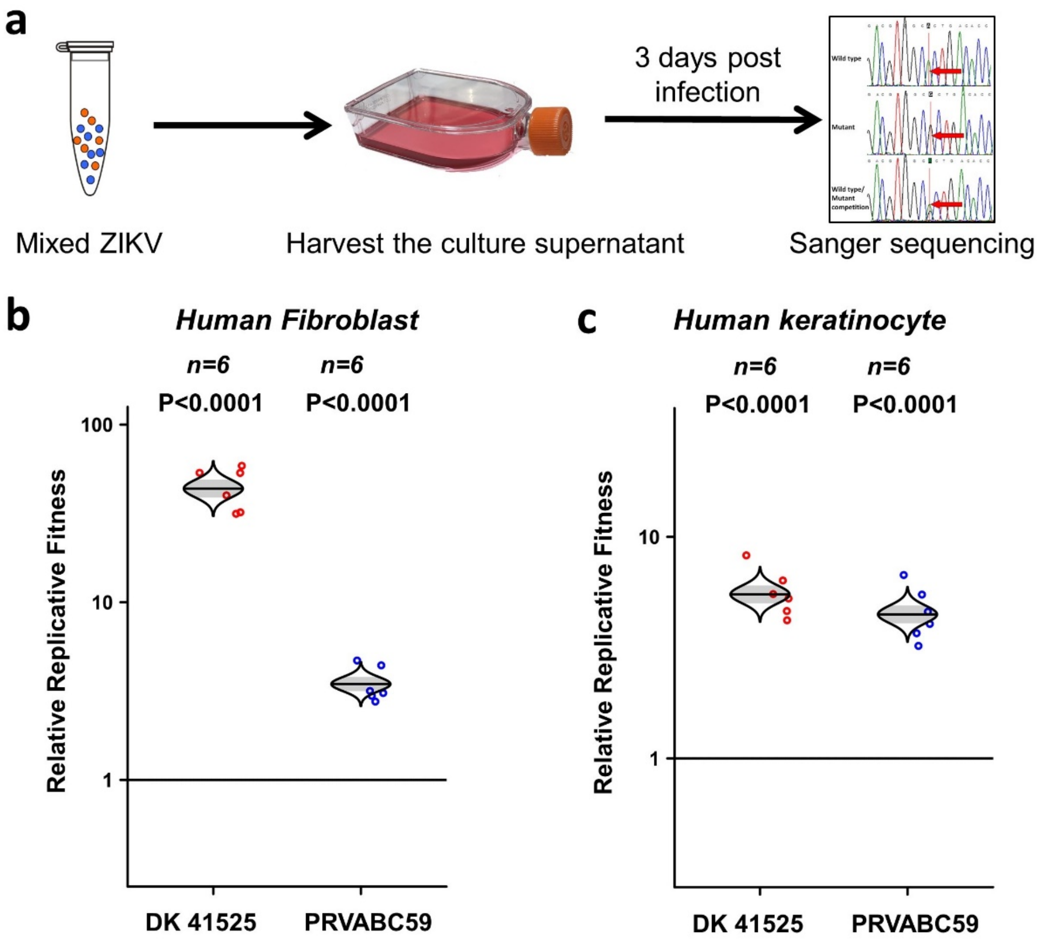

Supplementary Fig. 7. Fitness comparison of African and post-epidemic against a pre-epidemic ZIKV stain in human primary cells.

a, Schematic representation of the study design. The mixed ZIKVs were inoculated into human fibroblast and keratinocyte cells. The RNAs in the culture supernatant were isolated, amplified by RT-PCR, Sanger-sequenced 3 days post infection. **b**, **c**, The fitness of ZIKV African strain (Dakar 41525), Asian pre-epidemic (FSS13025) strain and American strain (PRVABC59) in human primary fibroblast (**b**) and keratinocyte (**c**) cells. Each point represents a single culture sample The distribution of the model-adjusted means is illustrated by catseye plots with shaded +/- standard error overlaid by scatterplots of subject measures; scatterplots have been randomly jittered horizontally for clarity, and are shown on the log (base-10) scale such that comparisons are against a null value of 1. *P* values are calculated for the group (strain) coefficient for each linear regression model. *n*=6 biologically independent samples from 2 independent repeats. Source data are provided as a Source Data file.

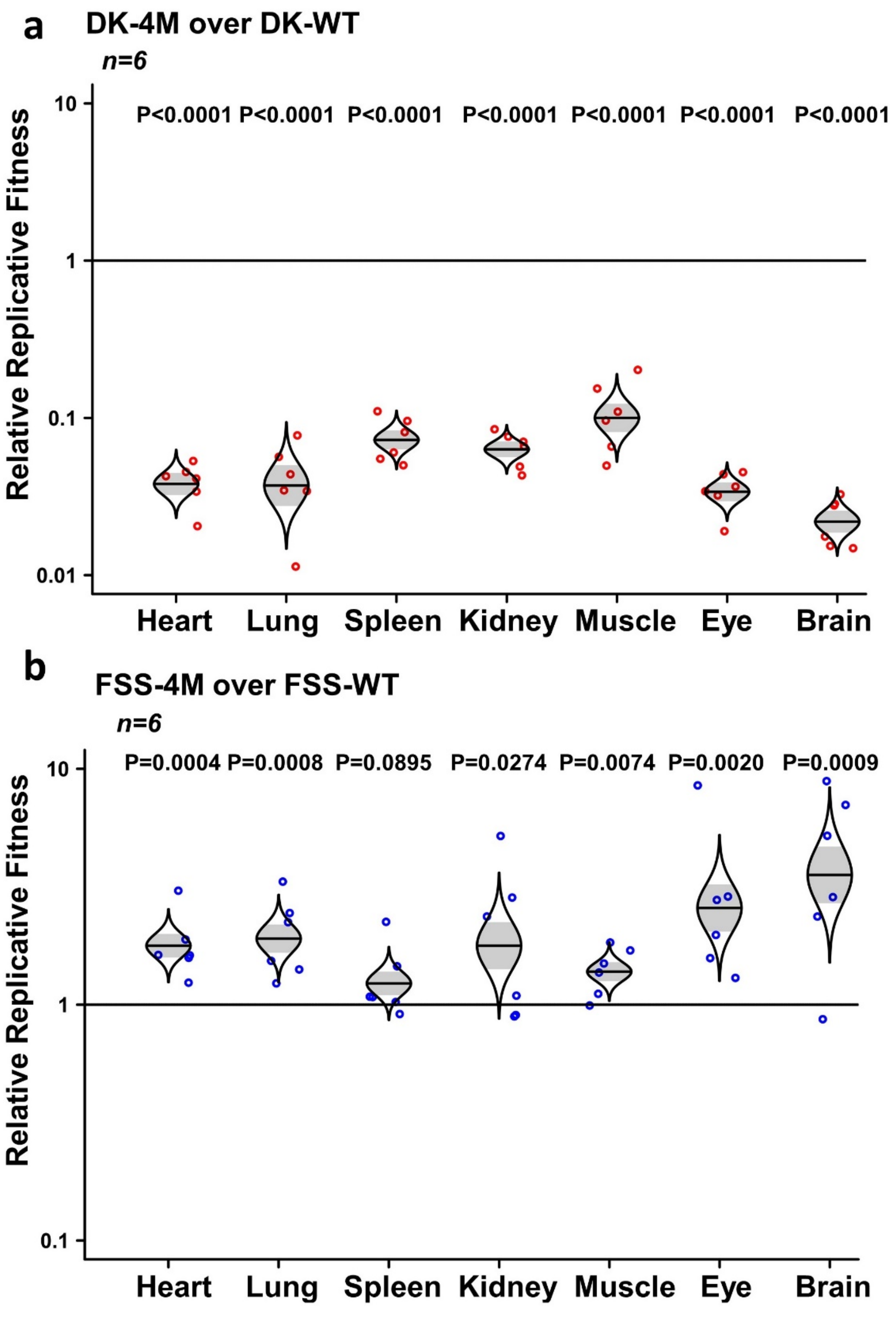

Supplementary Fig. 8. Fitness comparisons of Dakar-4 amino-acid mutant and FSS13025-4 amino-acid mutant against wild-type viruses in different organs of A129 mice.

a, Fitness comparison between Dakar 4-amino acid mutant (DK-4M) and the wild-type strain in different organs of A129 mice. **b**, The fitness comparison between FSS 4 amino-acid mutant (FSS-4M) and wild-type strain in different organs of A129 mice. Each point represents a single mouse sample. **a**, **b**, The distribution of the model-

adjusted means is illustrated by catseye plots with shaded +/- standard error overlaid by scatterplots of subject measures; scatterplots have been randomly jittered horizontally for clarity, and are shown on the log (base-10) scale such that comparisons are against a null value of 1. * $P < 0.05$, ** $P < 0.01$, *** $P < 0.001$, n.s. not significant. P values are calculated for the group (strain) coefficient for each linear regression model. n=6 biologically independent samples from a single experiment. Source data are provided as a Source Data file.

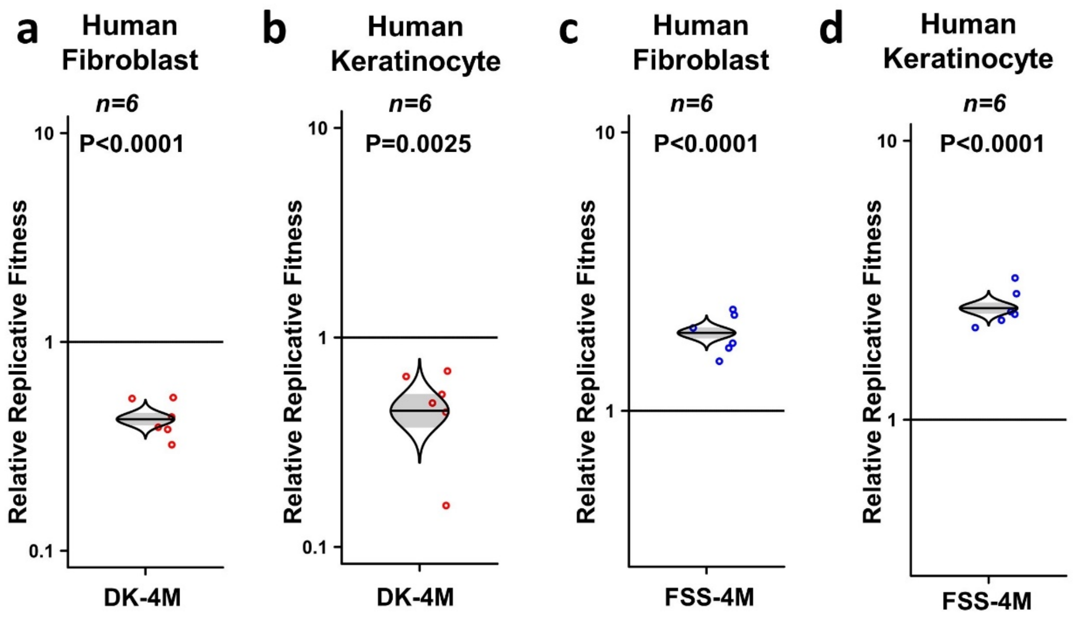

Supplementary Fig. 9. Fitness comparisons of Dakar-4 amino-acid mutant and FSS13025-4 amino-acid mutant versus wild-type ZIKV strains in human primary cells.

a, **b**, Fitness comparison between Dakar 4-amino acid mutant (DK-4M) and wild-type strain in human primary fibroblast (**a**) and keratinocyte (**b**) cells. **c**, **d**, The fitness comparison between FSS13025 4-amino acid mutant (FSS-4M) and wild-type strain in human primary fibroblast (**c**)and keratinocyte (**d**) cells. Each point represents a single culture sample. **a**-**d**, The distribution of the model-adjusted means is illustrated by catseye plots with shaded +/- standard error overlaid by scatterplots of subject measures; scatterplots have been randomly jittered horizontally for clarity, and are shown on the log (base-10) scale such that comparisons are against a null value of 1. *P* values are calculated for the group (strain) coefficient for each linear regression model. *n*=6 biologically independent samples from 2 independent repeats. Source data are provided as a Source Data file.

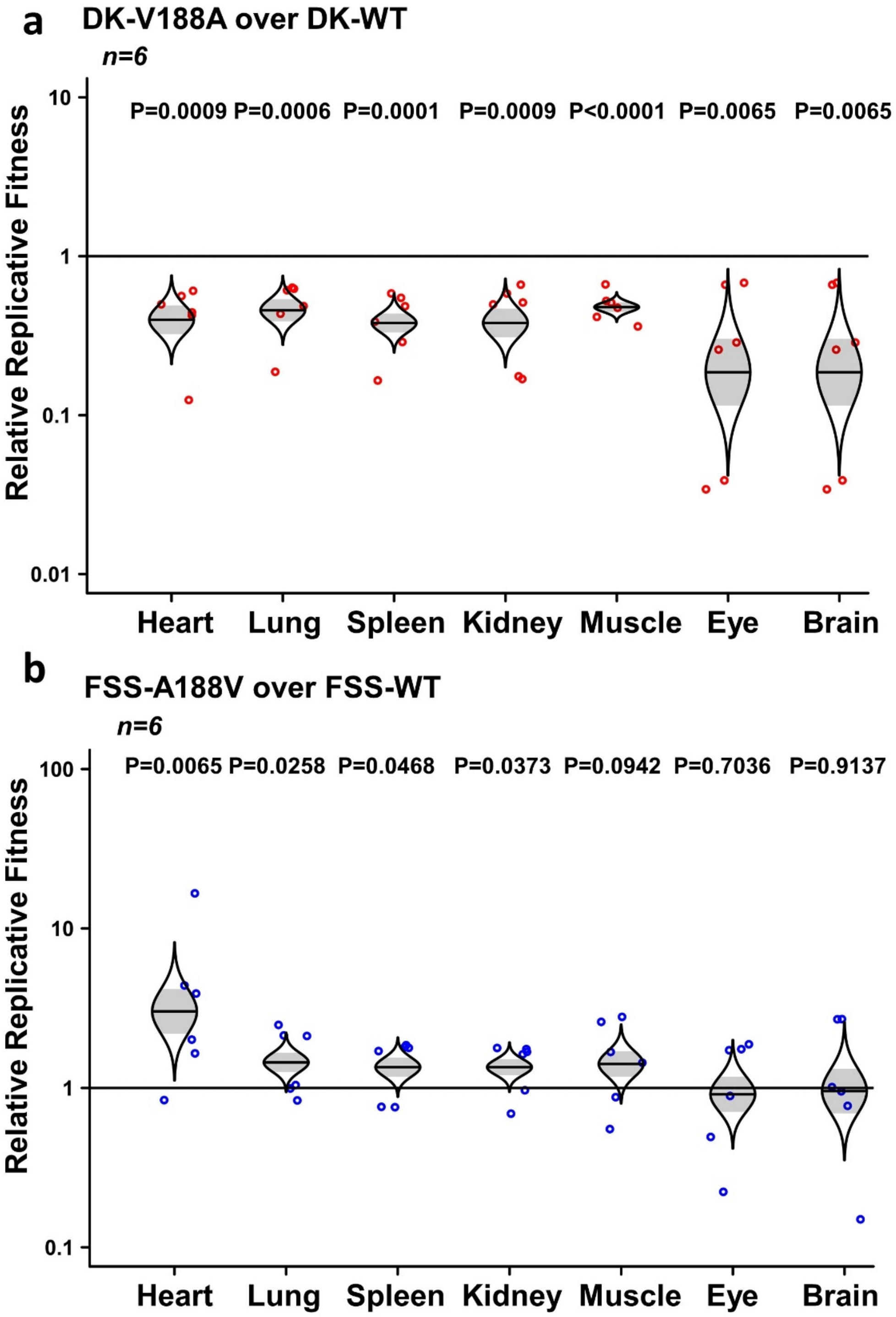

Supplementary Fig. 10. Fitness comparison between NS1-188 mutants against wild-type ZIKV strains in different organs of A129 mice.

a, Fitness comparison between Dakar NS1-V188A mutant and wild-type strain in different organs of A129 mice. **b**, Fitness comparison between FSS13025 NS1-A188V mutant and wild-type strain in different organs of A129 mice. Each point represents a single mouse sample **a**, **b**, The distribution of the model-adjusted means is illustrated

by catseye plots with shaded +/- standard error overlaid by scatterplots of subject measures; scatterplots have been randomly jittered horizontally for clarity, and are shown on the log (base-10) scale such that comparisons are against a null value of 1. *P* values are calculated for the group (strain) coefficient for each linear regression model. *n*=6 biologically independent samples from a single experiment. Source data are provided as a Source Data file.

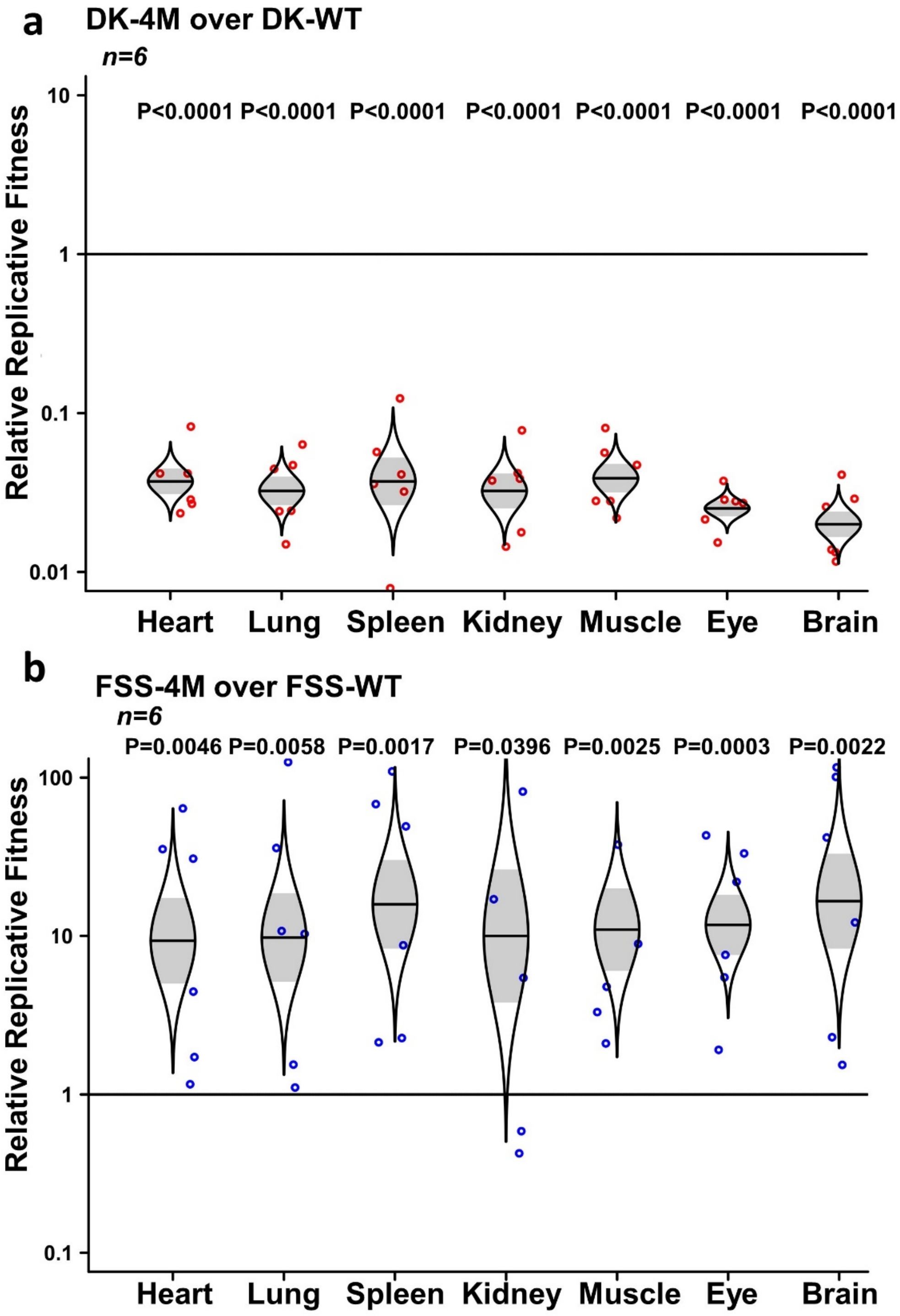

Supplementary Fig. 11. Fitness comparison between African-4 amino-acid mutant and FSS13025-4 amino-acid mutant against wild-type viruses during mosquito-mouse-mosquito transmission cycle.

a, Fitness comparison between Dakar 4-amino acid mutant (DK-4M) and wild-type strain in different organs of A129 mice after being bitten by infected mosquitoes. **b**, Fitness comparison between FSS 4-amino acid mutant (FSS-4M) and wild-type strain

in different organs of A129 mice biting by infected mosquitoes. Each point represents a single mosquito or mouse sample **a**, **b**, The distribution of the model-adjusted means is illustrated by catseye plots with shaded +/- standard error overlaid by scatterplots of subject measures; scatterplots have been randomly jittered horizontally for clarity, and are shown on the log (base-10) scale such that comparisons are against a null value of 1. *P* values are calculated for the group (strain) coefficient for each linear regression model. *n*=6 biologically independent samples from a single experiment. Source data are provided as a Source Data file.
