# Supplementary material for: Role of mutational reversions and fitness restoration in Zika virus spread to the Americas
Source: Nat Commun. 2021 Jan 26;12:595. doi: 10.1038/s41467-020-20747-3 (PMC7838395; doi:10.1038/s41467-020-20747-3)
Supplement: Supplementary file 3 — Description of Additional Supplementary Files [file 41467_2020_20747_MOESM3_ESM.docx]

**Supplementary Data 1.**

Representative Asian ZIKV strains with amino acid residues indicated for all positions shown to influence transmission efficiency or virulence.

**Supplementary Data 2**.

Phenotypes assigned to these amino acid substitutions are summarized.

**Supplementary Data 3.**

The relative replicative fitness of ZIKV mutant strains in mosquitoes, mice and human primary cells, derived from the data in the figures, are presented as the final ratio divided by the initial ratio of the two competing viruses, as used previously to compare adaptive chikungunya virus mutations^43^. P values are based on differences from equal fitness or a relative fitness value of 1.

**Supplementary Data 4.**

Primers and probes for gene cloning, qPCR, RT-PCR and Sanger sequencing.
