# Supplementary material for: Role of mutational reversions and fitness restoration in Zika virus spread to the Americas
Source: Nat Commun. 2021 Jan 26;12:595. doi: 10.1038/s41467-020-20747-3 (PMC7838395; doi:10.1038/s41467-020-20747-3)
Supplement: Supplementary file 8 — Reporting Summary [file 41467_2020_20747_MOESM8_ESM.pdf]

nature research

Corresponding author(s): Scott Weaver

Last updated by author(s): Dec 11, 2020

Reporting Summary

Nature Research wishes to improve the reproducibility of the work that we publish. This form provides structure for consistency and transparency in reporting. For further information on Nature Research policies, see our Editorial Policies and the Editorial Policy Checklist.

Statistics

For all statistical analyses, confirm that the following items are present in the figure legend, table legend, main text, or Methods section.

n/a	Confirmed	
☐	☒	The exact sample size (n) for each experimental group/condition, given as a discrete number and unit of measurement
☐	☒	A statement on whether measurements were taken from distinct samples or whether the same sample was measured repeatedly
☐	☒	The statistical test(s) used AND whether they are one- or two-sided<br>*Only common tests should be described solely by name; describe more complex techniques in the Methods section.*
☐	☒	A description of all covariates tested
☐	☒	A description of any assumptions or corrections, such as tests of normality and adjustment for multiple comparisons
☐	☒	A full description of the statistical parameters including central tendency (e.g. means) or other basic estimates (e.g. regression coefficient) AND variation (e.g. standard deviation) or associated estimates of uncertainty (e.g. confidence intervals)
☐	☒	For null hypothesis testing, the test statistic (e.g. F, t, r) with confidence intervals, effect sizes, degrees of freedom and P value noted<br>*Give P values as exact values whenever suitable.*
☐	☒	For Bayesian analysis, information on the choice of priors and Markov chain Monte Carlo settings
☐	☒	For hierarchical and complex designs, identification of the appropriate level for tests and full reporting of outcomes
☐	☒	Estimates of effect sizes (e.g. Cohen's d, Pearson's r), indicating how they were calculated

Our web collection on statistics for biologists contains articles on many of the points above.

Software and code

Policy information about availability of computer code

Data collection	The peak electropherogram height was collected using the QSVanalyser program(version 20121206).
Data analysis	Statistical analyses were performed using R statistical software (version 3.6.1),catseyes (version 0.2.3),Mesquite (version 3.6.1) and Graphad Prism (version 7.03).

For manuscripts utilizing custom algorithms or software that are central to the research but not yet described in published literature, software must be made available to editors and reviewers. We strongly encourage code deposition in a community repository (e.g. GitHub). See the Nature Research guidelines for submitting code & software for further information.

Data

Policy information about availability of data

All manuscripts must include a data availability statement. This statement should provide the following information, where applicable:

- Accession codes, unique identifiers, or web links for publicly available datasets
- A list of figures that have associated raw data
- A description of any restrictions on data availability

Extended Data and source data for generating main figures are available in the online version of the paper. Any other information is available upon request.

Field-specific reporting

Life sciences study design

All studies must disclose on these points even when the disclosure is negative.

Sample size	No statistical methods were used to pre-determine the sample size. Sample size was chosen based on previous experience and standards in the field.
Data exclusions	Mosquitoes that died before assay were excluded from analysis. The criteria were pre-established.
Replication	Competition experiments in mosquitoes and human primary cells were performed at least twice and were confirmed using different species; competition in mice were performed once using 6 animals and were confirmed using different methods. All attempts at replication were successful.
Randomization	Mosquitoes and mice were randomly allocated into different groups.
Blinding	The investigators were not blinded to the allocation during the experiments or to the outcome assessment. Blinding is not necessary because the results are quantitative and did not require subjective judgment or interpretation. Blinding is not typically used in the field.

Reporting for specific materials, systems and methods

We require information from authors about some types of materials, experimental systems and methods used in many studies. Here, indicate whether each material, system or method listed is relevant to your study. If you are not sure if a list item applies to your research, read the appropriate section before selecting a response.

Materials & experimental systems

n/a	Involved in the study	
☐	☒	Antibodies
☐	☒	Eukaryotic cell lines
☒	☐	Palaeontology and archaeology
☐	☒	Animals and other organisms
☒	☐	Human research participants
☒	☐	Clinical data
☒	☐	Dual use research of concern

Methods

n/a	Involved in the study	
☒	☐	ChIP-seq
☒	☐	Flow cytometry
☒	☐	MRI-based neuroimaging

Antibodies

Antibodies used	Mouse anti-ZIKV 4G2 primary antibody (MAB10216, Sigma), Goat anti-mouse secondary antibody(M8770, Sigma).
Validation	All antibodies were commercially available. See the corresponding manufacturer datasheets on webpages for reference and validation.

Eukaryotic cell lines

Policy information about cell lines

Cell line source(s)	Vero cells (CCL81) and C6/36 cells were purchased from the American Type Culture Collection. The human primary dermal fibroblast cells and human epidermal keratinocyte cells were purchased from Lonza.
Authentication	The Vero and C6/36 cells have been authenticated by ATCC through STR profiling; the human primary dermal fibroblast cells and human epidermal keratinocyte cells have been authenticated by Lonza through QC testing.
Mycoplasma contamination	All the cell lines we used were negative for mycoplasma contamination.
Commonly misidentified lines (See ICLAC register)	No commonly misidentified cell line was used.

Animals and other organisms

Policy information about studies involving animals; ARRIVE guidelines recommended for reporting animal research

Laboratory animals	The A129 mice, which are deficient in type I interferon receptors, 6-8 weeks of age, were used for the mouse study. The Aedes aegypti Rockefeller strain and A. aegypti Dominican Republic strain (F6), 7 days old, were used for mosquito experiments. Both male and female animals were used.
Wild animals	No wild animals were used in this study.

Field-collected samples	No field-collected samples were used in this study.
Ethics oversight	Mouse and mosquito studies were performed in accordance with the guidance for the Care and Use of Laboratory Animals of the University of Texas Medical Branch (UTMB). The protocol (protocol number 1708051 for mice) were approved by the Institutional Animal Care and Use Committee (IACUC) at UTMB. All the mouse manipulations were performed under anesthesia by isoflurane.

Note that full information on the approval of the study protocol must also be provided in the manuscript.
