# Supplementary material for: Role of mutational reversions and fitness restoration in Zika virus spread to the Americas
Source: Nat Commun. 2021 Jan 26;12:595. doi: 10.1038/s41467-020-20747-3 (PMC7838395; doi:10.1038/s41467-020-20747-3)
Supplement: Supplementary file 2 — Peer Review File [file 41467_2020_20747_MOESM2_ESM.pdf]

Editorial Note: This manuscript has been previously reviewed at another journal that is not operating a transparent peer review scheme. The manuscript was considered suitable for publication without further review at *Nature Communications.*
